# LTPNet: Lesion-Aware Triple-Path Feature Fusion Network for Skin Lesion Segmentation

**DOI:** 10.3390/jimaging12030093

**Published:** 2026-02-24

**Authors:** Yange Sun, Sen Chen, Huaping Guo, Li Zhang, Hongzhou Yue, Yan Feng

**Affiliations:** 1 School of Computer and Information Technology, Xinyang Normal University, Xinyang 464000, China; yangesun@xynu.edu.cn (Y.S.); chensen@xynu.edu.cn (S.C.); zhangli@xynu.edu.cn (L.Z.); yuehz@xynu.edu.cn (H.Y.); yfeng@xynu.edu.cn (Y.F.); 2 Henan Key Laboratory of Education Big Data Analysis and Application, Xinyang Normal University, Xinyang 464000, China

**Keywords:** skin lesion segmentation, attentive spatial modulator, lesion-aware lite-gate attention, triple-path feature fusion

## Abstract

Skin lesion segmentation has achieved notable progress in recent years; however, accurate delineation remains challenging due to complex backgrounds, ambiguous boundaries, and low lesion-to-skin contrast. To address these issues, we propose the lesion-aware triple-path feature fusion network (LTPNet), an end-to-end framework that progressively processes features through extraction, refinement, and aggregation stages. In the extraction stage, we incorporate a general foreground–background attention to suppress background interference and accelerate model convergence. In the refinement stage, we introduce an attentive spatial modulator (ASM) to jointly exploit local structural cues and global semantic context for precise spatial modulation. We further develop a lesion-aware lite-gate attention (LALGA) module that performs local spatial feature modulation and global channel recalibration tailored to lesion characteristics. In the aggregation stage, we propose a triple-path feature fusion (TPFF) module that explicitly models feature relationships across scales via three complementary pathways: a common path (CP) for semantic consistency, a saliency path (SP) for highlighting co-activated regions, and a difference path (DP) for accentuating structural discrepancies. Extensive experiments on in-domain and cross-domain datasets show that LTPNet achieves superior segmentation accuracy with reasonable inference efficiency and model complexity, demonstrating its potential for efficient and reliable clinical decision support.

## 1. Introduction

Skin cancer is among the most common and potentially lethal malignancies worldwide, and early diagnosis with timely intervention is essential to improve patient survival [1,2,3]. However, early skin lesions often exhibit subtle morphology, poorly defined boundaries, or low contrast, complicating timely detection [4]. Furthermore, in clinical practice, diagnosis relies heavily on dermatologists’ visual inspection and subjective judgment, which is time-consuming and prone to inter-observer variability, thereby increasing the likelihood of missed or incorrect assessments [5,6]. Consequently, automated skin lesion segmentation has become an urgent research focus within medical image analysis.

Traditional approaches to skin lesion segmentation, including edge-based techniques [7], clustering algorithms [8], and active-contour models [9], depend heavily on hand-crafted priors, limiting their robustness and generalizability across diverse lesion morphologies and noisy clinical data. In recent years, deep learning has demonstrated remarkable adaptability and powerful feature representation capabilities [10]. Among these methods, convolutional neural networks (CNNs) [11] have shown particularly strong performance, inspiring encoder–decoder architectures such as UNet [12], which leverage multiscale feature fusion and skip connections to preserve spatial details. To enhance feature representation and information flow, various techniques have been incorporated into UNet, including residual learning [13], dense connectivity [14], semantic-gap bridge [15], and adaptive skip connections [16]. The integration of Vision Transformers (ViTs) [17] into UNet architectures further enhances skin lesion segmentation by modeling long-range dependencies. For instance, TransUNet [18] embeds a ViT encoder to capture global context, complementing UNet’s inherent fine-grained localization and improving boundary delineation. CSWin-UNet [19] employs cross-shaped window attention to balance computational efficiency with segmentation accuracy. More recently, Mamba [20], a structured state-space model (SSM), has emerged as a promising alternative due to its ability to capture global contextual dependencies with linear computational complexity [21,22]. VM-UNet [5] pioneers the integration of Mamba modules into UNet architectures, while subsequent variants such as VMUNetv2 [23], ASP-VMUNet [4], DermoMamba [24], SkinMamba [25], and UltraLight-VMUNet [26] pursue enhanced multiscale feature extraction and global information utilization with sustained efficiency.

**Figure 1:**
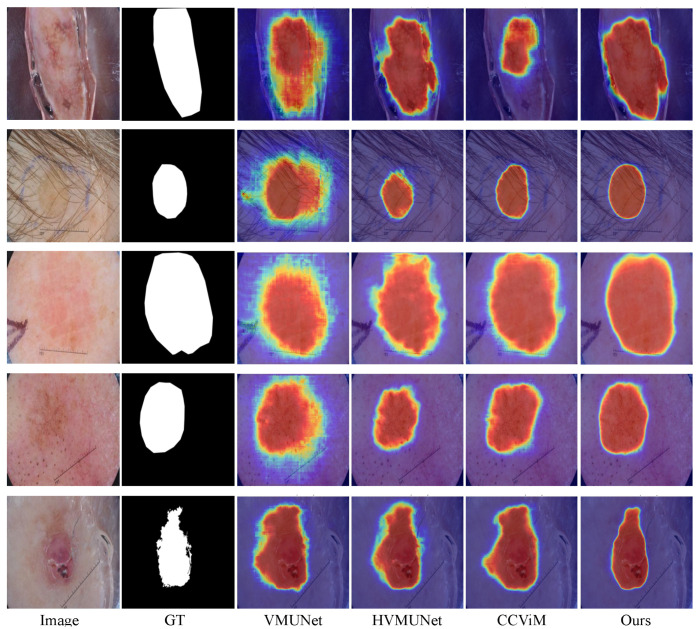
Segmentation heatmap visualizations of four methods on three types of challenging cases: complex background (Rows 1 and 2), low lesion-to-skin contrast (Rows 3 and 4), and blurred boundaries (Row 5).

Existing efforts in skin lesion segmentation have achieved impressive results, as discussed above; however, their performance remains limited in several challenging scenarios. As shown in Figure 1, HVMUNet [27], VMUNet [26], and CCViM [10] often fail to delineate accurate boundaries for lesions embedded in complex backgrounds, such as those with indistinct edges or hair interference (Rows 1 and 2). This limitation is further amplified under low skin–lesion contrast (Rows 3 and 4), where VMUNet, HVMUNet, and CCViM yield blurred boundary predictions. In cases where the boundary is inherently unclear (Row 5), the three representative methods exhibit noticeable over-segmentation.

To address the aforementioned limitations, we propose a lesion-aware triple-path feature fusion network (LTPNet) for skin lesion segmentation. LTPNet implements a progressive three-stage pipeline: feature extraction, refinement, and aggregation. In the feature extraction stage, we adopt a foreground-background attention (FBA) module that suppresses background interference while emphasizing lesion-relevant regions. In the feature refinement stage, we design two complementary modules: (1) an attentive spatial modulator (ASM) that employs parallel local attention (residual gated attention, RGA) and global selective scanning (2D Selective Scan, SS2D) pathways to enhance spatial coherence and boundary discrimination and (2) a lesion-aware lite-gate attention (LALGA) module that combines lightweight asymmetric gating attention (LAGA) for local spatial-guided feature modulation with global-guided channel recalibration (GGCA) to emphasize diagnostically relevant features. In the aggregation stage, we propose a triple-path feature fusion (TPFF) module that explicitly models cross-level feature interactions through three complementary pathways: the common path (CP), the saliency path (SP), and the difference path (DP). Specifically, CP enhances semantic consistency across feature levels through element-wise addition, SP captures co-occurrence patterns between low- and high-level features via multiplicative interaction, and DP highlights structural discrepancies and boundary-related variations through element-wise subtraction.

In summary, the main contributions of this paper are as follows:

- We propose an ASM that leverages dual pathways to capture overall lesion contours and fine boundary details, enhancing structural coherence and boundary discrimination.
- We design an LALGA that employs a lightweight gating mechanism together with context-guided channel recalibration to emphasize diagnostically relevant features and suppress background noise.
- We develop a TPFF that adaptively integrates three complementary pathways to capture consistent lesion semantics, emphasize boundary-sensitive regions, and enhance skin–lesion contrast through explicit modeling of multiscale feature relationships.
- Based on ASM, LALGA and TPFF, a novel segmentor called LTPNet is proposed. Extensive experiments on four publicly available datasets verify the effectiveness and generalization of LTPNet, demonstrating its competitive performance against SOTA methods.

The remainder of this paper is organized as follows. Section 2 reviews related work and motivation of our LTPNet. Section 3 describes our LTPNet, including ASM, LALGA, and TPFF. Section 4 presents experimental results and analyses. Section 5 discusses our method and potential future directions. Section 6 concludes this paper.

## 2. Related Work and Motivation

### 2.1. Evolution of Skin Lesion Segmentation

The challenge of skin lesion segmentation stems primarily from complex background interference, low contrast with surrounding tissues, and the highly irregular morphology of lesions. To address these issues, architectures tailored for skin lesion analysis have undergone a continuous evolution.

Early CNN-based models such as UNet [12,28] and its variants leverage encoder–decoder structures to extract multiscale features. However, CNNs inherently possess a limited receptive field, making it difficult to capture the long-range spatial dependencies of irregular skin lesions. Transformer-based designs [29,30] have been subsequently introduced, providing stronger global modeling capabilities. For example, TransUNet [18] integrates a Vision Transformer into UNet, but its quadratic complexity restricts scalability to high-resolution images.

Recently, Mamba [20], built on SSMs, has provided a new perspective for skin lesion segmentation by enabling long-sequence modeling via selective-scanning mechanisms with linear computational complexity. In this context, VM-UNet [5] is the first to integrate Mamba modules into a UNet backbone for skin lesion segmentation, while VMUNetv2 [23] further introduces semantic–detail fusion components tailored for lesion analysis. Building on these advances, subsequent Mamba-based approaches for skin lesions, including ASP-VMUNet [4], DermoMamba [24], SkinMamba [25], and UltraLight-VMUNet [26], have focused on enhancing multiscale feature extraction and global context modeling while maintaining computational efficiency for high-resolution skin lesion images.

Despite these advances, three major limitations persist: (1) Mamba-based models tend to prioritize global modeling while overlooking fine-grained local details that are essential for precise boundary delineation; (2) existing architectures lack dedicated refinement modules capable of enhancing lesion-specific features within complex backgrounds; and (3) current multiscale fusion strategies often rely on simple concatenation or addition, limiting their ability to capture the complementary and competitive relationships among hierarchical lesion features.

### 2.2. Attention Mechanisms and Feature Enhancement

Attention mechanisms reinforce feature discriminability by selectively emphasizing task-relevant regions through dynamic weighting. In skin lesion segmentation, their application has become increasingly diverse.

CNN-based approaches such as CSCA U-Net [31] highlight salient lesion areas via channel–spatial composite attention. MLK-Net [32] introduces mixed multi-head convolutions for multiscale representation learning, while HDS-Net [33] employs dynamic sparse attention to filter irrelevant responses and enhance subtle structural cues. Fractal U-Net [34] strengthens lesion feature representation through densely nested skip connections.

Transformer-based designs such as FAT-Net [35] extract global contextual cues via feature-adaptive attention, and SkinFormer [36] augments multiscale features by injecting statistical texture priors into Transformer blocks. Hybrid architectures including CCT-Net [37], FocalTransNet [38], and DSU-Net [39] combine CNN and Transformer modules to simultaneously model local detail and global semantics.

Despite this progress, two challenges remain prominent for skin lesion segmentation: (1) complex background interference that obscures lesion–background discrimination and (2) the tendency of informative responses to be submerged by noise. These issues require attention mechanisms capable of selective enhancement, noise suppression, and structural modeling.

### 2.3. Multiscale Feature Fusion Strategies

Effective multiscale fusion is crucial for enabling segmentation models to capture both global semantics and fine-grained boundaries. CNN-based architectures typically rely on skip connections to integrate multilevel features, while Mamba-based frameworks such as VMUNetv2 [23] enhance the interaction between low-level details and high-level semantics through dedicated semantic–detail fusion modules.

The underlying motivation of these techniques is to mitigate the loss of spatial detail incurred during downsampling. However, for skin lesions, features at different levels not only differ in resolution but also contain information that is inherently complementary and/or conflicting. Simple fusion strategies such as direct concatenation or element-wise addition may lead to suboptimal feature integration [40], as they treat multilevel features uniformly without considering their varying importance or semantic consistency.

To address these issues, recent studies have explored selective fusion strategies, such as attention-based weighting or gating mechanisms, which aim to adaptively regulate the contribution of multilevel features according to their contextual relevance. Although these methods introduce adaptive regulation into the fusion process, the fusion of multilevel lesion features remains non-trivial, highlighting the need for further refinement.

### 2.4. Motivation of This Work

Based on the above analysis, existing approaches for skin lesion segmentation still encounter three task-specific bottlenecks in skin lesion image analysis:

(a) **Inadequate Integration of Global Context and Local Detail**: Although hybrid backbones (e.g., MobileMamba [41]) combine local and global modeling capabilities, they lack a dedicated spatial refinement module designed to enhance intra-lesion consistency and boundary structures within multiscale features. Mamba-based models effectively capture global dependencies, but they tend to lose fine-grained local details that are essential for accurate boundary delineation in skin lesion images.
(b) **Limited Feature Discriminability Enhancement Mechanisms**: Most attention mechanisms focus on either channel-wise (e.g., SE [42]) or spatial-wise (e.g., BAM [43]) weighting, without providing a synergistic mechanism that jointly leverages local spatial cues and global contextual information to modulate feature representations. Existing dual-dimensional attention, such as the composite attention in CSCA U-Net [31], considers both dimensions but does not sufficiently incorporate the unique distribution characteristics of skin lesions, including texture irregularities, and background complexity.
(c) **Crude Modeling of Multiscale Fusion Relationships**: Mainstream fusion strategies (e.g., addition and concatenation operations) often fail to jointly capture complex interactions across hierarchical features, including region commonality, boundary discrepancies, and saliency reinforcement [44]. Consequently, the model struggles to characterize lesions with diverse morphologies and scales. Although VMUNetv2 [23] attempts semantic–detail fusion, it does not explicitly model the diverse interaction patterns specific to skin lesion structures.

To address these bottlenecks specific to skin lesion segmentation, we propose LTPNet, which incorporates three task-specific modules that systematically mitigate the above bottlenecks. Specifically, to address the first bottleneck, we introduce ASM, where parallel RGA and SS2D pathways are employed to reinforce spatial consistency and sharpen structural details, allowing the model to preserve boundary-sensitive local cues while retaining global contextual information. To alleviate the second bottleneck, we design LALGA, which integrates LAGA for local spatial-guided feature modulation and GGCA for capturing long-range contextual dependencies. By jointly leveraging local spatial cues and global channel statistics reflecting skin lesion distribution patterns, LALGA enhances the discriminative power of features. To overcome the third bottleneck, we design TPFF, which explicitly models cross-level feature interactions through three parallel pathways, i.e., CP, SP, and DP, enabling refined and discriminative feature aggregation for skin lesion segmentation.

## 3. Methods

### 3.1. Overall Architecture

**Figure 2:**
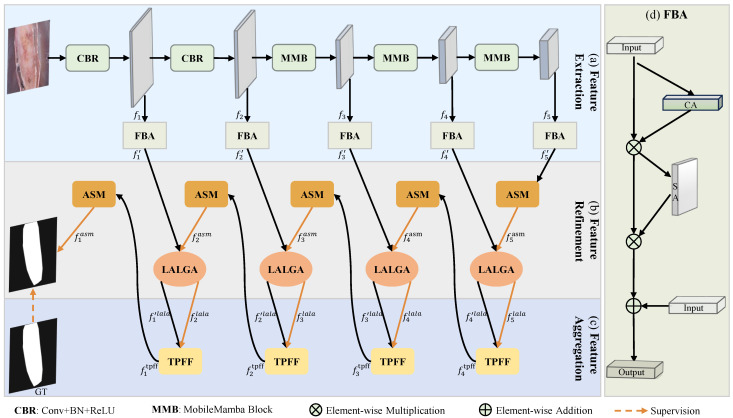
Overview of our LTPNet architecture, comprising three stages: (**a**) feature extraction, (**b**) feature refinement, and (**c**) feature aggregation, which collaboratively produce the final segmentation. (**d**) Illustration of the foreground–background attention (FBA) module.

We propose LTPNet, a novel architecture that sequentially performs feature extraction, refinement, and aggregation for skin lesion segmentation. The overall framework is illustrated in Figure 2.

**Feature Extraction:** We adopt MobileMamba as the backbone, leveraging its dual modeling strengths: convolutional locality for fine structural details and Mamba for efficient long-range context capture. As shown in Figure 2a, given an input image $x\in R^{H\times W\times C}$, the backbone extracts five hierarchical features ${\left\{f_{i}\right\}}_{i=1}^{5}$, where $f_{i}$ denotes the feature at the *i*th level with spatial dimensions $H_{i}=\frac{H}{2^{i+1}}$, $W_{i}=\frac{W}{2^{i+1}}$, and channel count $C_{i}$. To address the challenge of complex background interference in skin lesion images, we integrate a foreground–background attention (FBA) module [45] at each scale of MobileMamba. As illustrated in Figure 2d, FBA sequentially applies channel-based attention and spatial-based attention to emphasize lesion-relevant regions while suppressing background noise. Formally,

(1) $$f_{i}^{\prime }=\mathrm{FBA}\left(f_{i}\right)=\mathrm{SA}\left(\mathrm{CA}\left(f_{i}\right)\right)⊕f_{i},\;i\in \left\{1,2,3,4,5\right\},$$

where ⊕ denotes element-wise addition.

**Feature Refinement:** This stage processes complementary inputs from the extraction and aggregation stages to enhance both spatial structures and channel-wise semantics across network levels, as shown Figure 2b. To achieve this purpose, we introduce the ASM and LALGA modules, which are applied sequentially within the refinement stage. ASM employs parallel local and global branches to capture boundary details and holistic context, respectively, producing structure-aware intermediate features. Based on the spatially refined features generated by ASM, LALGA performs fine-grained adaptation via a lightweight asymmetric gating mechanism and context-guided channel recalibration, emphasizing salient semantic patterns while suppressing background noise at the channel level. The refinement process is formally defined as:

(2) $$\begin{array}{cc} & f_{i}^{\mathrm{asm}}=\left\{\begin{array}{cccc} & \mathrm{ASM}\left(f_{i}^{\prime }\right), & & \mathrm{if}i=5,\\ & \mathrm{ASM}\left(f_{i}^{\mathrm{tpff}}\right), & & \mathrm{if}i=\{1,2,3,4\}, \end{array}\right. \end{array}$$

(3) $$\begin{array}{cc} & f_{i}^{\mathrm{lala}}=\mathrm{LALGA}\left(f_{i}^{\mathrm{asm}}\right),i\in \left\{2,3,4,5\right\}, \end{array}$$

(4) $$\begin{array}{cc} & f_{i}^{\prime \mathrm{lala}}=\mathrm{LALGA}\left(f{’}_{i}\right),i\in \left\{1,2,3,4\right\}, \end{array}$$

where $f_{i}^{\mathrm{tpff}}$ denotes the output of the feature aggregation stage at the *i*-level. Through ASM and LALGA, the refinement stage progressively yields discriminative and boundary-consistent representations specifically tailored for skin lesion characteristics.

**Feature Aggregation:** To address the crude modeling of multiscale feature relationships in existing skin lesion segmentation methods, we introduce TPFF, which explicitly characterizes complementary cross-level interactions while integrating the enhanced representations produced by the feature refinement stage. Specifically, at the *i*th level (i∈{1,2,3,4}), TPFF takes two inputs: (1) the refined feature $f_{i}^{\prime \mathrm{lala}}$ obtained from the same level through feature extraction and subsequent enhancement and (2) the higher-level enhanced representation $f_{i+1}^{\mathrm{lala}}$ generated at the (i+1)th level and further refined by ASM. TPFF then performs adaptive multiscale fusion between these two inputs to produce a consolidated representation. Formally,

(5) $$f_{i}^{\mathrm{tpff}}=\mathrm{TPFF}\left(f_{i+1}^{\mathrm{lala}},f_{i}^{\prime \mathrm{lala}}\right),i\in \left\{1,2,3,4\right\}.$$

Finally, $f_{1}^{\mathrm{tpff}}$ is processed by ASM to produce the final segmentation mask.

The mainly innovative components of our LTPNet include ASM, LALGA and TPFF, and the corresponding details are presented in Section 3.2, Section 3.3 and Section 3.4.

### 3.2. Attentive Spatial Modulator (ASM)

Skin lesions frequently exhibit irregular boundaries and heterogeneous internal textures, necessitating a mechanism capable of capturing fine-grained local structures while simultaneously preserving global shape consistency. Therefore, we propose ASM, which dynamically modulates feature responses by jointly exploiting localized structural cues and holistic semantic context, enabling the network to emphasize homogeneous lesion regions while suppressing background interference commonly observed in skin lesion images.

**Figure 3:**
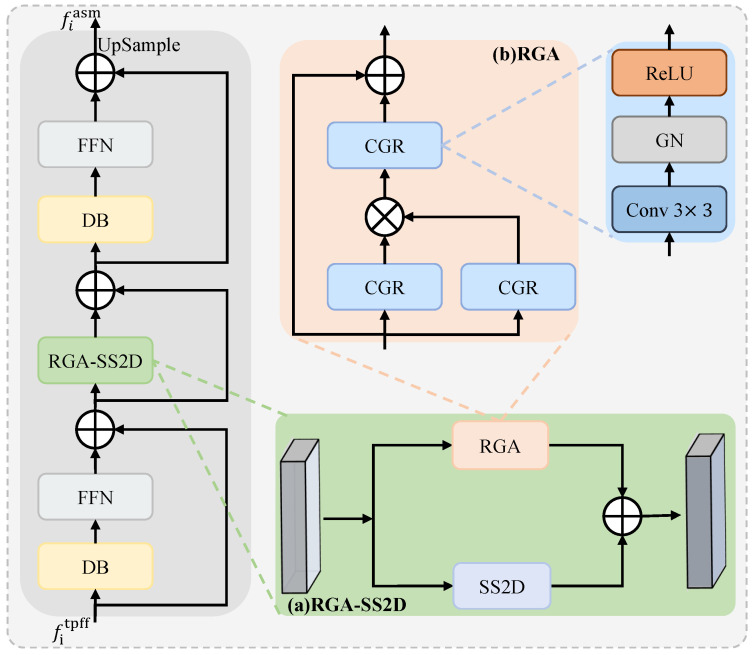
Structure of the proposed ASM, including (**a**) RGA-SS2D, is designed to adaptively enhance the spatial coherence and saliency of lesion-related features. (**b**) llustration of the RGA module.

The structure of ASM is illustrated in Figure 3. The key component of ASM is the RGA-SS2D unit, which operates on the aggregated representation $f_{i}^{\mathrm{tpff}}$ (with the deepest level using $f_{5}^{\mathrm{tpff}}=f_{5}^{\prime }$). In this unit, RGA enhances localized structural cues, whereas SS2D [46] captures long-range global dependencies. The computation is formulated as follows:

(6) $$\begin{array}{cc} & {\hat{f}}_{i}^{1}=\mathrm{FFN}\left(\mathrm{DB}\left(f_{i}^{\mathrm{tpff}}\right)\right)+f_{i}^{\mathrm{tpff}}, \end{array}$$

(7) $$\begin{array}{cc} & {\hat{f}}_{i}^{2}=\mathrm{RGA}\text{-}\mathrm{SS}2D\left({\hat{f}}_{i}^{1}\right)+{\hat{f}}_{i}^{1}, \end{array}$$

(8) $$\begin{array}{cc} & {\hat{f}}_{i}^{3}=\mathrm{FFN}\left(\mathrm{DB}\left({\hat{f}}_{i}^{2}\right)\right)+{\hat{f}}_{i}^{2}, \end{array}$$

(9) $$\begin{array}{cc} & f_{i}^{\mathrm{asm}}=\mathrm{UpSample}\left({\hat{f}}_{i}^{3}\right), \end{array}$$

where DB(·) denotes a 3×3 depthwise separable convolution with batch normalization, FFN(·) is a lightweight feedforward block for nonlinear refinement, and UpSample(·) applies bilinear interpolation.

Figure 3a shows the structure of RGA-SS2D, which processes the input feature ${\hat{f}}_{i}^{1}$ in two parallel branches:

(10) $$\begin{array}{c} \mathrm{RGA}\text{-}\mathrm{SS}2D\left({\hat{f}}_{i}^{1}\right)=\mathrm{SS}2D\left({\hat{f}}_{i}^{1}\right)+\mathrm{RGA}\left({\hat{f}}_{i}^{1}\right), \end{array}$$

where the SS2D [46] pathway focuses on global context modeling. Specifically, the feature ${\hat{f}}_{i}^{1}$ is unfolded along four structured scanning paths into 1D sequences, passed through a content-aware selective scanning mechanism, and then reshaped back into 2D form, producing multi-directional global semantic cues that facilitate precise modeling of lesion structures.

Figure 3b shows the structure of the RGA module, which performs adaptive feature recalibration by employing two parallel CGR branches to extract complementary representations from ${\hat{f}}_{i}^{1}$. Each CGR consists of a 3×3 convolution, group normalization (GN), and a ReLU activation. The outputs of the two branches are combined via element-wise multiplication, allowing the module to dynamically emphasize salient lesion features while suppressing redundant responses:

(11) $${\hat{f}}_{i}^{\prime 1}=\mathrm{CGR}\left({\hat{f}}_{i}^{1}\right)⊗\mathrm{CGR}\left({\hat{f}}_{i}^{1}\right),$$

where ⊗ denotes element-wise multiplication. The resulting feature ${\hat{f}}_{i}^{\prime 1}$ is further refined through an additional CGR block to enhance sensitivity to small and irregular lesion structures. Finally, a residual shortcut is applied to preserve the original input and maintain feature continuity:

(12) $$\mathrm{RGA}\left({\hat{f}}_{i}^{1}\right)=\mathrm{CGR}\left({\hat{f}}_{i}^{\prime 1}\right)+{\hat{f}}_{i}^{1}.$$

### 3.3. Lesion-Aware Lite-Gate Attention (LALGA)

**Figure 4:**
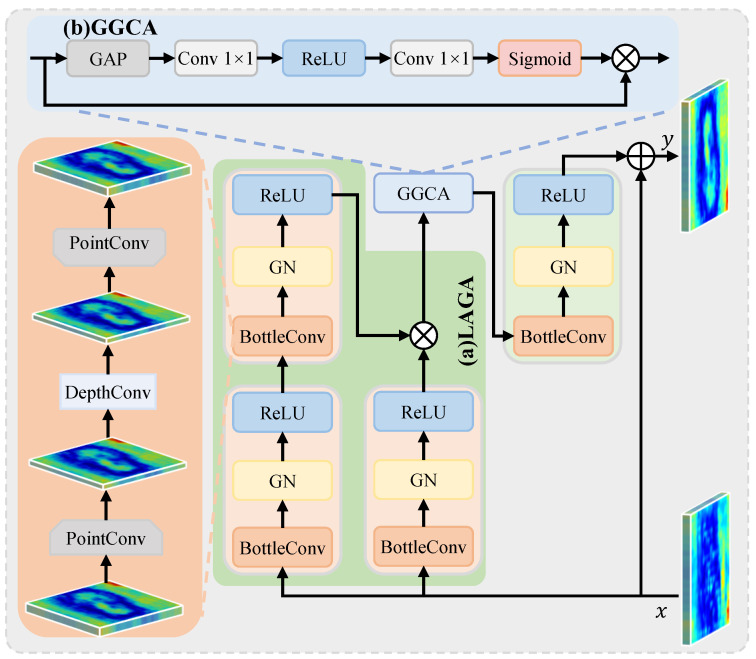
Structure of the proposed LALGA, comprising (**a**) LAGA and (**b**) GGCA, is designed to emphasize discriminative feature channels.

Skin lesion segmentation is often disturbed by background interference, with effective lesion features often submerged by noise. To address these issues, we design an LALGA that leverages spatial lesion cues to strengthen discriminative feature channels. As illustrated in Figure 4, LALGA consists of two complementary components: LAGA for local spatially guided feature modulation and GGCA for capturing long-range contextual dependencies. Given the input feature *x*, which can be either $f_{i}^{\prime }$ from the extraction stage or $f_{i+1}^{\mathrm{asm}}$ from the aggregation stage (see Figure 2), the overall process is as follows:

(13) $$y=\mathrm{BGR}\left(\mathrm{GGCA}\left(\mathrm{LAGA}\left(x\right)\right)\right)+x,$$

where BGR(·) denotes a bottleneck convolution (BottleConv) followed by group normalization (GN) and ReLU activation.

#### 3.3.1. Lightweight Asymmetric Gating Attention (LAGA)

We propose an LAGA module that enhances local lesion features by leveraging critical spatial information. As illustrated in Figure 4a, LAGA adopts an asymmetric dual-path structure in which one path preserves the primary feature representation, whereas the other employs a deeper transformation to generate spatially adaptive gating weights for feature modulation.

Specifically, given an input feature *x*, LAGA processes it through two branches. The shallow branch applies a single BGR block to extract the base feature $g_{1}$, while the deep branch cascades two BGR blocks to produce the gating feature $g_{2}$:

(14) $$\begin{array}{cc} g_{1} & =\mathrm{BGR}(x), \end{array}$$

(15) $$\begin{array}{cc} g_{2} & =\mathrm{BGR}(\mathrm{BGR}(x)). \end{array}$$

The outputs of both branches are fused via element-wise multiplication $f_{\mathrm{gate}}=g_{1}⊗g_{2}$, where $g_{2}$ functions as the attention weight to modulate $g_{1}$. By asymmetrically allocating computational depth, the shallow path preserves spatial detail precision, while the deeper path further enhances critical spatial features, thereby improving feature discriminability. In this way, LAGA achieves a favorable trade-off between computational cost and representational capability for skin lesion feature enhancement.

#### 3.3.2. Global-Guided Channel Attention (GGCA)

LAGA modulates feature representations via spatially adaptive gating driven by local spatial information, thereby emphasizing lesion-relevant regions and suppressing background noise, as discussed in Section 3.3.1. However, LAGA may overlook the global spatial characteristics and holistic contextual distribution, which potentially lead to inconsistencies in channel activation. To address this limitation, we design a GGCA that models global contextual relationships through adaptive channel recalibration, as illustrated in Figure 4b.

GGCA takes $f_{\mathrm{gate}}$, the LAGA output, as input and performs global average pooling (GAP) along spatial dimensions to obtain a compact channel descriptor encoding global context statistics. This descriptor is then processed by two successive 1×1 convolution layers with a ReLU activation inserted between them to capture nonlinear inter-channel dependencies, followed by a sigmoid activation to generate channel-wise attention weights *w*. Afterward, the weights *w* are applied to $f_{\mathrm{gate}}$ through element-wise multiplication, yielding the recalibrated feature $f_{\mathrm{gate}}^{\prime }$:

(16) $$\begin{array}{cc} & w=\sigma (\mathrm{Conv}\left(\mathrm{ReLU}\left(\mathrm{Conv}\left(\mathrm{GAP}\left(f_{gate}\right)\right)\right)\right)), \end{array}$$

(17) $$\begin{array}{cc} & f_{gate}^{\prime }=f_{gate}⊗w, \end{array}$$

where σ(·) denotes sigmoid activation.

Using global context for channel recalibration, GGCA strengthens discriminative channels that contain critical lesion information while suppressing channels dominated by background noise or redundancy. This global modulation complements LAGA’s local spatial guidance, resulting in more consistent and context-aware representations.

### 3.4. Triple-Path Feature Fusion (TPFF)

To reconcile cross-level feature discrepancies and enhance lesion discriminability, we propose TPFF that explicitly models complementary interactions among multiscale features through three parallel pathways, namely CP, SP, and DP. This design is motivated by the observation that features at different resolutions often contain inherently complementary and/or conflicting information for skin lesion representation, requiring sophisticated fusion beyond simple concatenation or summation.

**Figure 5:**
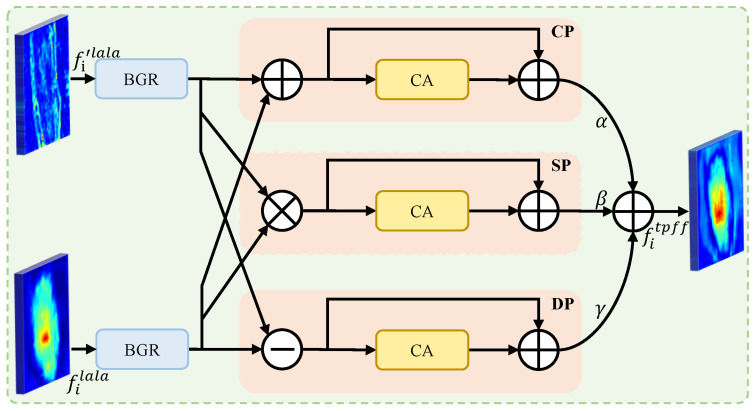
Structure of the proposed TPFF, comprising CP, SP, and DP, designed to reconcile cross-level feature discrepancies and enhance lesion discriminability.

As illustrated in Figure 5, TPFF takes the features $f_{i}^{\prime \mathrm{lala}}$ and $f_{i+1}^{\mathrm{lala}}$ from the refinement stage as input, where i∈{1,2,3,4}, enabling hierarchical feature aggregation across scales (see Figure 2). Each input is first processed independently by a BGR block, which consists of a 3×3 bottleneck convolution followed by group normalization and a ReLU activation, producing detail-preserving representations:

(18) $$\begin{array}{c} f_{\mathrm{low}}=\mathrm{BGR}\left(f_{i}^{\prime \mathrm{lala}}\right), \end{array}$$

(19) $$\begin{array}{c} f_{\mathrm{high}}=\mathrm{BGR}\left(f_{i+1}^{\mathrm{lala}}\right). \end{array}$$

The two features $f_{\mathrm{low}}$ and $f_{\mathrm{high}}$ are then jointly fed into CP, SP, and DP pathways to capture complementary interactions from different perspectives. The outputs of these three pathways are adaptively integrated to form the fused representation:

(20) $$\begin{array}{cc} f_{i}^{\mathrm{tpff}}= & \alpha \mathrm{CP}\left(f_{\mathrm{low}},f_{\mathrm{high}}\right)+\beta \mathrm{SP}\left(f_{\mathrm{low}},f_{\mathrm{high}}\right)\\ & +\gamma \mathrm{DP}(f_{\mathrm{low}},f_{\mathrm{high}}), \end{array}$$

where α=0.4,β=0.2 and γ=0.4 are learnable coefficients that balance the contributions of the three pathways. These coefficients are constrained to satisfy α+β+γ=1, ensuring balanced contributions from all pathways at the start of training and promoting stable gradient updates.

#### 3.4.1. Common Path (CP)

CP adopts a simple yet effective additive fusion strategy to integrate complementary information from adjacent scales:

(21) $$f_{\mathrm{cp}}=f_{\mathrm{low}}⊕f_{\mathrm{high}}.$$

This linear aggregation promotes semantic consistency across feature levels while preserving the overall feature distribution, thereby providing a stable and scale-aligned representation that alleviates discrepancies introduced by resolution differences.

The aggregated feature $f_{\mathrm{cp}}$ is subsequently processed by a channel attention (CA) module [42], which adaptively recalibrates channel-wise responses to emphasize lesion-relevant semantic cues. To further maintain representational continuity and avoid excessive feature distortion, a residual shortcut is incorporated:

(22) $$f_{\mathrm{cp}}^{\mathrm{ca}}=\mathrm{CA}\left(f_{\mathrm{cp}}\right)+f_{\mathrm{cp}}.$$

#### 3.4.2. Saliency Path (SP) and Difference Path (DP)

In parallel with CP, SP and DP further enrich multiscale feature fusion by modeling complementary cross-level interactions from two distinct perspectives. Unlike CP that focuses on semantic alignment through additive aggregation, SP and DP explicitly account for scale-wise agreement and discrepancy, respectively. Specifically, SP employs multiplicative interaction to capture co-occurrence patterns between low- and high-level features, thereby highlighting spatial locations where both scales exhibit strong activations. In contrast, DP performs differential modeling through element-wise subtraction to extract structural discrepancies and accentuate boundary-related variations that are suppressed in shared responses. Formally,

(23) $$\begin{array}{cc} f_{\mathrm{sp}} & =f_{\mathrm{low}}⊗f_{\mathrm{high}}, \end{array}$$

(24) $$\begin{array}{cc} f_{\mathrm{dp}} & =f_{\mathrm{low}}⊖f_{\mathrm{high}}, \end{array}$$

where ⊖ denotes element-wise subtraction.

To maintain consistency with CP and enhance the discriminative capability of both pathways, the intermediate features $f_{\mathrm{sp}}$ and $f_{\mathrm{dp}}$ are refined by a CA module, followed by residual enhancement to preserve the fidelity of the original low-level representation:

(25) $$\begin{array}{cc} f_{\mathrm{sp}}^{\mathrm{ca}} & =\mathrm{CA}\left(f_{\mathrm{sp}}\right)+f_{\mathrm{low}}, \end{array}$$

(26) $$\begin{array}{cc} f_{\mathrm{dp}}^{\mathrm{ca}} & =\mathrm{CA}\left(f_{\mathrm{dp}}\right)+f_{\mathrm{low}}. \end{array}$$

### 3.5. Loss Function

To ensure stable optimization and accurate boundary delineation, we adopt a hybrid loss that combines the binary cross-entropy (BCE) [47] and Dice [48] losses:

(27) $$\begin{array}{cc} & L_{\mathrm{BceDice}}=\lambda_{1}\cdot L_{\mathrm{Bce}}+\lambda_{2}\cdot L_{\mathrm{Dice}}, \end{array}$$

where $\lambda_{1}$ and $\lambda_{2}$ balance the relative contributions of the two terms and are set to 1 by default. BCE promotes accurate pixel-wise classification, while Dice mitigates background interference and enhances boundary delineation. The combination of these two losses facilitates stable convergence and improves segmentation performance across lesions of various shapes and sizes.

## 4. Experiments

### 4.1. Implementation Setup

#### 4.1.1. Datasets

We evaluate the performance of our LTPNet on four benchmark datasets, including PH2 [49], ISIC16 [50], ISIC17 [51], and ISIC18 [52].

**PH2:** The PH2 dataset contains 200 skin lesion images with region-based annotations and the corresponding segmentation masks. Following common practice, the dataset is split into 140 training images and 60 testing images.

**ISIC16, ISIC17 and ISIC18:** The international skin imaging collaboration (ISIC) datasets from 2016, 2017, and 2018 are widely used benchmark datasets for skin lesion segmentation. The ISIC16 dataset follows the official splits, comprising 900 training images and 379 testing images. Since the ISIC17 and ISIC18 datasets do not provide publicly available test sets and exhibit diverse baselines, we follow the data preparation protocols of MALUNet [53] and EGE-UNet [54], splitting both datasets into training and testing sets with a 7:3 ratio. Accordingly, ISIC17 contains 1,500 training images and 650 testing images, while ISIC18 comprises 1,886 training images and 808 testing images.

#### 4.1.2. Implementation Details

Our LTPNet is implemented in PyTorch 2.1.0 and trained on a NVIDIA A100 80 G GPU. AdamW [55] is adopted as the optimizer, with an initial learning rate set to 1e-3, which is gradually decayed using a predefined cosine annealing schedule. This schedule is fully specified before training and does not depend on any validation or test set feedback, ensuring smooth decay and avoiding potential information leakage. All images from the four datasets are uniformly resized to 256 × 256 pixels using bilinear interpolation to preserve texture and edge details, with corresponding masks resized via nearest neighbor interpolation, and processed in batches of 32 [5]. During training, data augmentation techniques [23], including random flipping and rotation, modify the images’ orientation to increase data diversity without changing the total number of images, helping to alleviate overfitting.

#### 4.1.3. Evaluation Metrics

To comprehensively evaluate the performance of LTPNet, we employ several standard evaluation metrics, including mean intersection over union (mIoU), Dice similarity coefficient (DSC), accuracy (Acc), specificity (Spe), and sensitivity (Sen) [2,5]. These metrics jointly assess segmentation quality from multiple perspectives, covering both region-level overlap and pixel-wise classification performance, as well as the balance between false positives and false negatives, which is particularly important for skin lesion image analysis. In addition, threshold-dependent evaluation curves, namely precision–recall (PR) and receiver operating characteristic (ROC) curves, are also reported [56].

### 4.2. Comparison with the SOTA

#### 4.2.1. Quantitative Comparison

**Figure 6:**
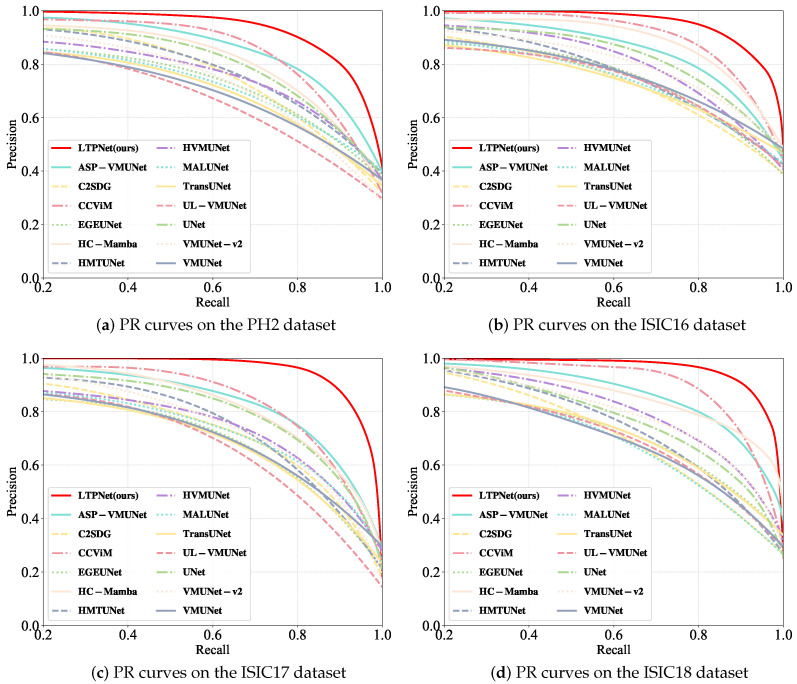
Precision–recall (PR) curves of our LTPNet and thirteen SOTA methods on the PH2, ISIC16, ISIC17, and ISIC18 datasets.

**Figure 7:**
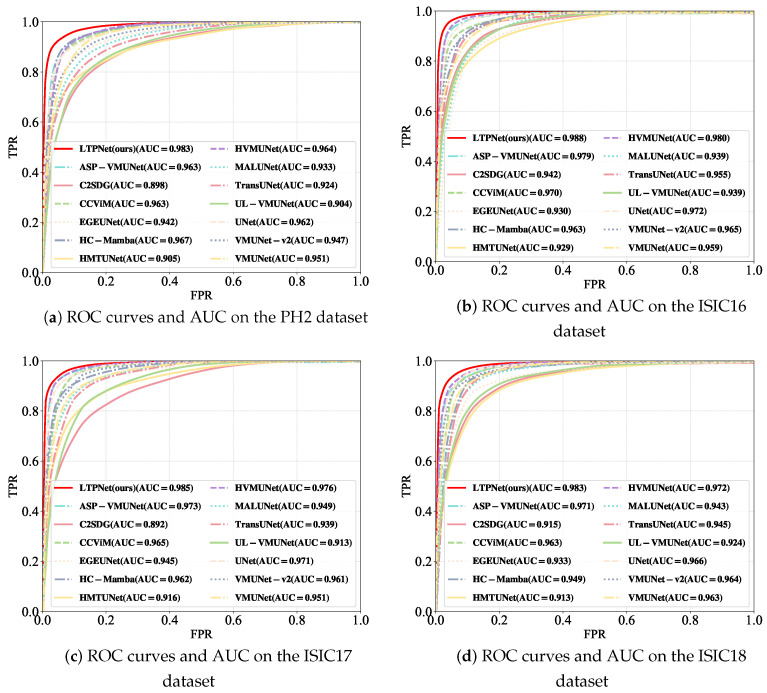
ROC curves and the corresponding area under the curve (AUC) on the PH2, ISIC16, ISIC17, and ISIC18 datasets.

We evaluate our LTPNet against thirteen SOTA methods, including four CNN-based approaches (UNet [12], MALUNet [53], EGEUNet [54], and C2SDG [57]), one Transformer-based method (TransUNet [18]), and eight Mamba-based approaches (UL-VMUNet [26], HMTUNet [58], HVMUNet [27], VMUNet [5], VMUNet-v2 [23], ASP-VMUNet [4], HC-Mamba [59], and CCViM [10]) on the PH2, ISIC16, ISIC17, and ISIC18 datasets. Table 1, Table 2, Table 3 and Table 4 report the quantitative comparison results on these datasets, respectively. The best performing method is highlighted in bold, while the second best result is underlined. In addition, Figure 6 and Figure 7 illustrate the corresponding precision–recall (PR) and receiver operating characteristic (ROC) curves for all compared methods across the four datasets. For a fair comparison, we run the publicly available source code of all competing methods with the default settings.

From Table 1, our LTPNet achieves the best overall performance on the PH2 dataset across mIoU, DSC, Acc, and Spe. Specifically, LTPNet obtains the highest mIoU (92.52%) and DSC (96.12%), reflecting accurate region overlap and strong boundary consistency. LTPNet also reaches the highest Acc (97.69%), demonstrating a reliable pixel-level classification. In addition, LTPNet achieves the highest Spe (98.22%), exceeding UNet’s 97.78%, and ranks second in Sen (96.43%), only slightly below TransUNet’s 96.52%, indicating a well-balanced trade-off between lesion recall and false positive suppression. Figure 6a and Figure 7a further support these observations. The PR curves in Figure 6a show that LTPNet retains higher precision across a wide range of recall levels, demonstrating stable lesion recognition under varying thresholds. Meanwhile, the ROC curves in Figure 7a reveal consistently higher TPRs at nearly all FPR levels, confirming its strong sensitivity–specificity balance.

On the ISIC16 dataset (Table 2), our LTPNet maintains superior performance, ranking first in mIoU (87.22%), DSC (93.18%), and Acc (96.14%), while achieving competitive Spe (97.21%) and Sen (93.41%). Although slightly surpassed by CCViM in Spe (97.40%) and by ASP-VMUNet in Sen (94.09%), LTPNet still offers a strong balance between false positive suppression and lesion recall, consistent with its behavior on the PH2 dataset. Figure 6b and Figure 7b corroborate these results: the PR curve of LTPNet (Figure 6b) exhibits consistently higher precision across a broad recall spectrum, while the ROC curve of LTPNet (Figure 7b) demonstrates superior TPRs at most FPR levels.

Table 3 and Table 4 demonstrate that our LTPNet continues to deliver top-tier performance on both ISIC17 and ISIC18, achieving the highest scores in mIoU, DSC, Acc, Spe, and Sen. Specifically, on ISIC17, LTPNet obtains mIoU of 81.04% (2.39% higher than CCViM), DSC of 89.53% (1.48% higher than CCViM), Acc of 96.55% (0.50% higher than CCViM), Spe of 98.26% (0.09% higher than TransUNet), and Sen of 88.04% (0.59% higher than HVMUNet). On ISIC18, LTPNet achieves mIoU of 82.54% (2.22% higher than ASP-VMUNet), DSC of 90.43% (1.34% higher than ASP-VMUNet), Acc of 95.34% (0.79% higher than CCViM), Spe of 96.91% (0.22% higher than HC-Mamba), and Sen of 90.47% (1.11% higher than VMUNet). Moreover, the PR and ROC curves on ISIC17, shown in Figure 6c and Figure 7c, respectively, and those on ISIC18, shown in Figure 6d and Figure 7d, consistently demonstrate that LTPNet maintains higher precision across varying recall levels and achieves superior true positive rate–false positive rate (TPR–FPR) characteristics compared with competing methods.

#### 4.2.2. Visual Comparison

**Figure 8:**
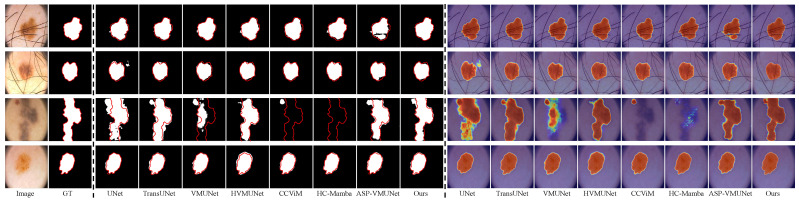
Visualization results of LTPNet and other SOTA methods on the PH2 dataset for three types of cases: complex backgrounds (Rows 1 and 2), indistinct lesion boundaries (Row 3), and low lesion–skin contrast (Row 4). Red contours indicate the ground truth boundaries.

**Figure 9:**
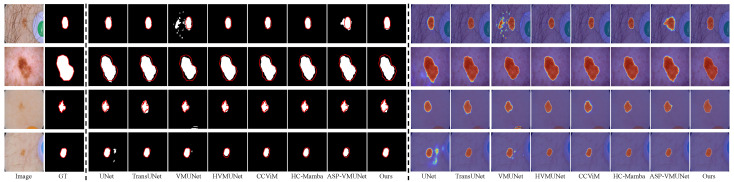
Visualization results of LTPNet and other SOTA methods on the ISIC16 dataset for three types of cases: complex backgrounds (Rows 1 and 2), indistinct lesion boundaries (Row 3), and low lesion–skin contrast (Row 4). Red contours indicate the ground truth boundaries.

**Figure 10:**
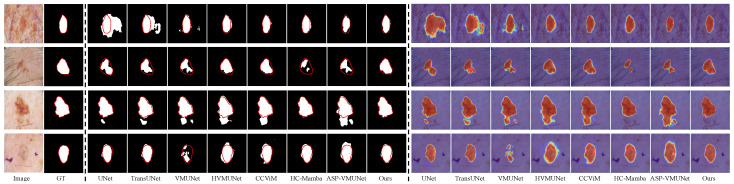
Visualization results of LTPNet and other SOTA methods on the ISIC17 dataset for three types of cases: complex backgrounds (Rows 1 and 2), indistinct lesion boundaries (Row 3), and low lesion–skin contrast (Row 4). Red contours indicate the ground truth boundaries.

**Figure 11:**
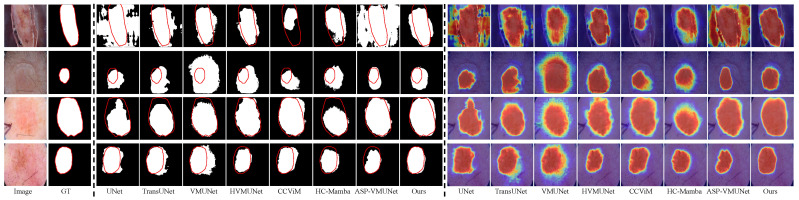
Visualization results of LTPNet and other SOTA methods on the ISIC18 dataset for three types of cases: complex backgrounds (Rows 1 and 2), indistinct lesion boundaries (Row 3), and low lesion–skin contrast (Row 4). Red contours indicate the ground truth boundaries.

We visually compare our LTPNet with seven SOTA methods, i.e., UNet, TransUNet, VMUNet, HVMUNet, CCViM, HC-Mamba, and ASP-VMUNet. Figure 8, Figure 9, Figure 10 and Figure 11 present qualitative comparisons on the PH2, ISIC16, ISIC17, and ISIC18 datasets. Each figure is arranged in 18 columns and 4 rows, where Columns 1 and 2 display the original images and their corresponding ground truth (GT) masks, Columns 3 to 10 show segmentation results, and Columns 11 to 18 depict the corresponding heatmaps. The four rows illustrate various challenging scenarios: Rows 1 and 2 contain complex backgrounds, Row 3 shows blurry boundaries, and Row 4 exhibits low skin–lesion contrast.

From Figure 8, our LTPNet demonstrates superior segmentation performance and generates more informative response heatmaps on the PH2 dataset. For complex backgrounds, such as illumination variations and hair occlusion (Rows 1 and 2), ASP-VMUNet produces fragmented segmentations within lesion areas, whereas TransUNet, HVMUNet, CCViM, and HC-Mamba yield overly smoothed lesion contours, losing fine-grained structural information. In comparison, our LTPNet maintains boundary continuity and preserves detailed lesion textures, producing segmentation results that closely match the ground truth masks. For blurred boundaries (Row 3), several baseline models, including VMUNet and TransUNet, fail to fully capture the lesion region, often producing incomplete or overly smoothed segmentations. CCViM and HC-Mamba fail to identify the lesion entirely in this scenario. For low skin–lesion contrast (Row 4), HVMUNet produces blurred boundaries and exhibits noticeable edge errors. Our LTPNet accurately delineates lesions with sharp, continuous boundaries, preserves subtle textures, and produces heatmaps with strong lesion activation and minimal background interference.

Similar results are observed from Figure 9, Figure 10 and Figure 11 for the ISIC16, ISIC17, and ISIC18 datasets, respectively. From Figure 9, under hair occlusion and complex backgrounds (Row 1), baseline models such as VMUNet and ASP-VMUNet produce fragmented or over-segmented lesions, while UNet yields sharp but incomplete boundaries under skin spot interference (Row 2). For blurred boundaries (Row 3) and low lesion–skin contrast (Row 4), competing models either under- or over-segment lesions and often lose fine structural details. As shown in Figure 10, baseline models, including UNet, TransUNet, and VMUNet, tend to over- or under-segment lesions under skin spot interference (Row 1) and hair occlusion (Row 2), and produce incomplete or inaccurate segmentations for blurred boundaries (Row 3) and low skin–lesion contrast (Row 4). Figure 11 shows that under complex backgrounds (Row 1), UNet and ASP-VMUNet tend to over-segment lesions, and the baseline model also exhibits pronounced over-segmentation under similar conditions (Row 2), and for blurred boundaries (Row 3) and low skin–lesion contrast (Row 4), baseline models either under- or over-segment lesions and lose fine details. By comparison, across all three datasets (ISIC16, ISIC17, and ISIC18), our LTPNet consistently produces precise and continuous lesion boundaries, preserves fine structural details, and generates focused heatmaps highlighting lesion regions, demonstrating its robustness and effectiveness in diverse challenging scenarios.

#### 4.2.3. Running Efficiency

We further evaluate the computational overhead and inference speed of our LTPNet against thirteen representative models, using an NVIDIA A100 80G GPU to ensure a fair comparison. As shown in Table 5, our LTPNet achieves strong segmentation performance while maintaining moderate model parameters, computational cost, and inference speed. This favorable balance between accuracy and efficiency demonstrates that LTPNet effectively reconciles high segmentation performance with practical computational requirements, making it suitable for clinical skin lesion analysis.

### 4.3. Ablation Study

#### 4.3.1. Structure Ablation

We conduct comprehensive ablation studies to evaluate the impact of the main components of our LTPNet, including FBA, ASM, LALGA, and TPFF. The corresponding results are summarized in Table 6, Table 7, Table 8 and Table 9 for the PH2, ISIC16, ISIC17, and ISIC18 datasets, respectively, where the model without any of our proposed techniques in LTPNet is regarded as the baseline (line 1). For fair comparison, we run the publicly available source code with default parameter settings. To ensure the reliability of the results, all experiments are conducted five times with different random seeds, and the mean and standard deviation for each metric are reported in the tables.

From Table 6, the baseline achieves mIoU, DSC, Acc, Spe, and Sen of 91.01%, 95.25%, 96.11%, 97.73%, and 95.02%, respectively, on the PH2 dataset. Introducing FBA leads to improvements in mIoU, DSC, and Sen, reaching 91.24%, 95.59%, and 95.23%, respectively, indicating its effectiveness in enhancing lesion–background distinction. Incorporating ASM further improves mIoU, Acc, Spe, and Sen to 91.99%, 96.61%, 98.01%, and 95.72%, respectively, highlighting its role in preserving structural information. Adding LALGA improves DSC and Acc to 95.92% and 97.37%, respectively, indicating its effectiveness in refining discriminative feature representations using spatial lesion cues. Finally, integrating TPFF yields the best performance, with mIoU, DSC, Acc, Spe, and Sen reaching 92.52%, 96.12%, 97.69%, 98.22%, and 96.43%, respectively. Additionally, removing FBA while retaining ASM, LALGA, and TPFF lowers the mIoU to 92.23%, indicating that FBA plays a key role in suppressing background interference and enabling the full synergy of the proposed modules.

Similar results are observed from Table 7, Table 8 and Table 9, indicating that each component consistently contributes to performance improvement across datasets. Specifically, on the ISIC16 dataset (Table 7), the baseline achieves mIoU, DSC, Acc, Spe, and Sen of 86.72%, 92.62%, 95.64%, 96.78%, and 92.89%, respectively. Incorporating FBA brings marginal gains (mIoU +0.03%, DSC −0.14%, Acc +0.12%, Spe +0.11%, Sen −0.02%), while the subsequent integration of ASM, LALGA, and TPFF further improves these metrics by 0.47%, 0.70%, 0.38%, 0.32%, and 0.54%, respectively. Removing FBA while keeping ASM, LALGA, and TPFF lowers mIoU to 86.82% (−0.40%), showing that FBA remains important for suppressing background interference and enabling module synergy. On the ISIC17 dataset (Table 8), the sequential incorporation of FBA, ASM, LALGA, and TPFF yields more notable improvements, increasing mIoU, DSC, Acc, and Sen by 2.47%, 1.53%, 0.45%, and 1.11%, respectively. A similar trend is also observed on the ISIC18 dataset (Table 9), where our LTPNet, equipped with all modules, attains the best performance, reaching 82.50%, 90.42%, 95.34%, 96.92%, and 90.46% in terms of mIoU, DSC, Acc, Spe, and Sen, respectively.

#### 4.3.2. TPFF Path Ablation

Table 10 presents the impact of different TPFF path combinations on segmentation performance evaluated on the ISIC18 dataset. Using only CP, the mIoU, DSC, Acc, Spe, and Sen are 82.12%, 90.13%, 95.09%, 96.79%, and 90.01%, respectively. Incorporating SP in parallel with CP increases the mIoU by 0.20%, DSC by 0.37%, Acc by 0.10%, and Sen by 0.22%. Incorporating DP alongside CP and SP achieves the highest overall performance, with most metrics improved except for a slight drop in DSC. These results demonstrate that each TPFF path contributes positively to segmentation, collectively enhancing both accuracy and structural consistency in lesion delineation.

#### 4.3.3. Effectiveness of LTPNet Modules

To further validate that the performance improvements originate from our proposed modules rather than the backbone itself, we conduct a comparison under the same MobileMamba backbone [41] across different methods. As shown in Table 11, the baseline model achieves 91.01% mIoU, 95.25% DSC, 96.11% Acc, 97.73% Spe, and 95.02% Sen. VMUNet with the MobileMamba improves these metrics to 91.77% mIoU, 95.71% DSC, 97.45% Acc, 97.97% Spe, and 96.20% Sen, while VMUNet-v2 further increases performance to 92.20% mIoU, 95.94% DSC, 97.59% Acc, 98.21% Spe, and 96.14% Sen. HMTUNet shows relatively lower results, with 90.24% mIoU, 94.87% DSC, 96.96% Acc, 97.86% Spe, and 94.82% Sen. By incorporating all proposed LTPNet modules, our LTPNet achieves the highest performance, reaching 92.52% mIoU, 96.12% DSC, 97.69% Acc, 98.22% Spe, and 96.43% Sen. These results indicate that the strong performance of LTPNet is not only attributable to the MobileMamba backbone but is also consistently enhanced by the proposed LTPNet modules.

### 4.4. Generalization Experiment

To evaluate the generalization capability of our LTPNet, we conduct comparisons with 13 representative methods on the CVC-ClinicDB dataset [60], a benchmark for colorectal polyp segmentation comprising 612 colonoscopy images with a resolution of 384 × 288 extracted from 31 video sequences. All images are uniformly resized to 256 × 256 and divided into training and validation sets with an 8:2 ratio. Experimental settings, including hyperparameters, optimization strategies, and evaluation metrics, follow the procedures described in Section 4.1.2 and Section 4.1.3. Table 12 presents the corresponding results.

As shown in Table 12, our LTPNet achieves the best performance in terms of mIoU (89.58%), DSC (94.51%), and Spe (99.55%) on the CVC-ClinicDB dataset, demonstrating its strong capability in accurately delineating polyp regions with diverse sizes and irregular shapes. Although VMUNet-v2 slightly outperforms LTPNet in Acc (99.08% vs. 99.08%, tied) and Sen (95.64% vs. 94.00%), the differences are marginal. Notably, LTPNet achieves a higher specificity, suggesting fewer false positives and more reliable background suppression. Overall, these results indicate that LTPNet achieves a favorable trade-off among segmentation accuracy, boundary precision, and false positive control, highlighting its strong generalization ability and robustness in challenging environments.

## 5. Discussion

Despite advances in skin lesion segmentation through deep learning, performance remains limited in challenging scenarios with complex backgrounds, blurred boundaries, and low contrast. We identify the key limitation not only in the backbone features, but more critically in the underexplored stages of feature refinement and aggregation. To address these issues, we propose LTPNet, a framework that enhances the backbone feature via FBA and performs explicit feature refinement and multi-perspective fusion through ASM, LALGA, and TPFF.

**Figure 12:**
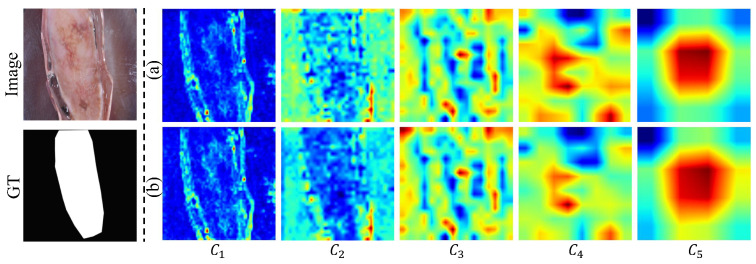
Visualization of feature extraction on the ISIC18 dataset. (**a**) MobileMamba; (**b**) MobileMamba equipped with the proposed FBA module. $C_{i}$ denotes the hierarchical feature extracted from the backbone network at the *i*th level.

Shallow backbone features generally retain rich spatial details but often include irrelevant background or artifacts, whereas deep features capture semantic information at the cost of fine-grained details. As shown in Figure 12a, shallow features ${\left\{C_{i}\right\}}_{i=1}^{3}$ preserve abundant boundary information but are affected by background interference, while deep features ${\left\{C_{i}\right\}}_{i=4}^{5}$ provide more abstract semantic cues. To mitigate this issue, we integrate FBA [45] to strengthens foreground regions while suppressing background interference by explicitly modeling channel-spatial attention between foreground and background areas. Figure 12b demonstrates that FBA enhances boundary details in shallow features and enriches semantic representation in deep features.

Heterogeneous feature responses often arise within the same skin lesion due to irregular shapes, size variations, and imaging noise. These inconsistencies disrupt spatial coherence among pixels belonging to the same anatomical structure, particularly those separated by long distances, leading to blurred or fragmented lesion boundaries. To address this problem, we propose ASM, a refinement module that adaptively enhances the spatial coherence and saliency of lesion-related features. Unlike conventional refinement strategies that rely solely on local filtering or apply uniform attention to all pixels, ASM jointly models local structural cues and global contextual dependencies through two complementary branches: an RGA branch, which employs residual gated attention to amplify fine-grained boundary details and suppress noise, and an SS2D branch, which performs selective scanning along multiple spatial directions to integrate long-range contextual information. By merging these dual perspectives, ASM enhances spatial coherence within lesion regions, ensuring consistent feature activation and preserving structural integrity in heterogeneous lesion morphologies.

Complementary to ASM, which refines spatial coherence, we design LALGA to further enhance feature semantics from the channel dimension. The skin lesions often exhibit irregular shapes, uneven distribution, and background interference, leading to inconsistent feature responses and blurred boundaries. Traditional attention mechanisms often apply uniform channel weighting or rely solely on global context, failing to comprehensively characterize lesions. To address this issue, LALGA leverages spatial saliency to modulate channel responses for more lesion-aware feature recalibration. Specifically, LALGA enhances lesion-relevant features through a dual-stage process: LAGA employs a lightweight asymmetric gating attention to perform local spatial-guided feature modulation, and GGCA performs a global-guided channel attention to capture long-range contextual dependencies.

Skin lesions often exhibit low contrast against surrounding healthy skin, irregular morphology, and heterogeneous texture, which can cause conventional feature fusion methods, such as simple concatenation or linear addition, to produce inconsistent or suboptimal representations. These traditional strategies fail to reconcile cross-level feature discrepancies and often under-utilize the complementary information present in multiscale representations. To address these problems, we propose a TPFF that explicitly models complementary feature interactions via a triple-path fusion strategy. Specifically, CP enhances cross-level semantic coherence by aggregating semantic information from low- and high-level features; SP highlights locally salient activations, preserving fine-grained details crucial for accurate lesion representation; and DP emphasizes skin–lesion contrast to improve boundary delineation and mitigate background interference. Unlike existing fusion methods that treat multilevel features uniformly or rely on single-path attention, TPFF adaptively integrates these three complementary perspectives through learnable coefficients, allowing the network to balance global context, local saliency, and boundary contrast in a unified manner.

**Figure 13:**
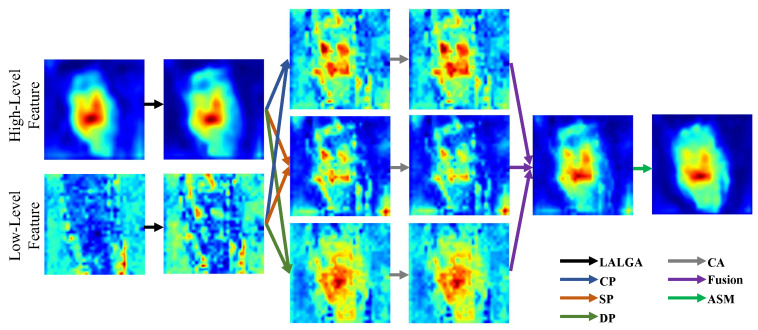
Visualization of the outputs of ASM, LALGA, and TPFF during the feature refinement and aggregation stages reveals each module’s contributions to enhancing feature representation and facilitating information fusion.

Figure 13 illustrates the visual impact of the proposed LALGA, TPFF, and ASM modules. Compared to baseline features, LALGA-enhanced features show stronger activations in lesion regions, particularly in areas with weak contrast or subtle textures, highlighting previously underrepresented local details. Following TPFF aggregation, lesion regions exhibit improved coherence and sharper boundaries: the CP pathway maintains global consistency, SP emphasizes salient local features, and DP strengthens the contrast between lesions and surrounding healthy skin. These effects collectively produce more precise and continuous segmentation cues, as observed in clearer lesion contours and more uniform activation patterns. Finally, ASM further refines the aggregated features by enhancing edge details and activating fine-grained textures, resulting in heatmaps with highly discriminative lesion representations and reduced background interference.

Extensive experiments validate the effectiveness of LTPNet and its key components while maintaining reasonable computational efficiency. However, we observe that LTPNet occasionally misclassifies a small number of background pixels as lesions (Figure 11, Rows 1 and 2). Possible reasons include: (1) ASM relies on SS2D rather than self-attention [29] for long-range dependency modeling, which may offer less precise global contextual cues in complex backgrounds; (2) LALGA’s saliency-guided channel enhancement may cause slight over-activation in low-contrast regions, allowing subtle background textures to be misinterpreted as lesion signals; and (3) TPFF may introduce minor fusion inconsistencies when CP, SP, and DP generate divergent responses in cases with highly irregular lesion boundaries, occasionally yielding weak activations near lesion–background transition zones.

Future work will focus on addressing the above limitations from three perspectives. First, to mitigate the reduced precision of global context modeling in ASM, we plan to replace SS2D with a lightweight long-range dependency modeling mechanism or develop a hybrid global–local attention design to enhance robustness in complex backgrounds. Second, to alleviate over-activation induced by LALGA in low-contrast regions, we aim to incorporate uncertainty-aware saliency estimation to stabilize channel responses and suppress noise-sensitive activations. Third, for TPFF, we intend to design an adaptive path-weighting strategy to dynamically reconcile discrepancies among CP, SP, and DP under challenging lesion morphologies, thereby improving fusion reliability and reducing false positive activations near lesion–background transitions. Additionally, we plan to investigate the potential of our refined segmentation maps to facilitate downstream classification using advanced classifiers, including ensemble learning methods [61], potentially enhancing overall diagnostic accuracy.

## 6. Conclusions

Skin lesion segmentation remains challenging due to complex backgrounds, indistinct lesion boundaries, and low skin–lesion contrast. This paper proposes an innovative LTPNet that aims to address these challenges from three perspectives: feature extraction, feature refinement, and feature aggregation. From the feature extraction perspective, a general FBA is employed to suppress background interference and guide the network to focus on candidate lesion regions, providing a robust initial representation. For feature refinement, we propose ASM and LALGA to enhance lesion features in a spatial–channel complementary manner: ASM strengthens spatial coherence and boundary perception by jointly modeling local structural cues and long-range context, while LALGA leverages saliency-guided channel recalibration to selectively emphasize lesion-relevant channels and suppress noise-sensitive or irrelevant activations. For feature aggregation, we propose TPFF to adaptively integrate hierarchical features by reconciling discrepancies among its three complementary paths, thereby achieving consistent global–local representation, enhancing lesion contrast, and preserving fine structural details. Extensive experiments on four public skin lesion datasets, as well as a colorectal polyp dataset, show that our LTPNet consistently achieves SOTA performance across multiple evaluation metrics, demonstrating strong robustness and adaptability to diverse lesion appearances and complex backgrounds.

## Figures and Tables

**Table 1:** Comparison of different models of the PH2 dataset. Bold values represent the best results, while underlined values represent the second-best results. ‘↑’ indicates that higher values are better.

Model	mIoU (%) ↑	DSC (%) ↑	Acc (%) ↑	Spe (%) ↑	Sen (%) ↑
UNet [12]	88.66	93.99	96.46	97.78	93.34
TransUNet [18]	90.89	92.53	97.14	97.40	**96.52**
MALUNet [53]	87.08	93.09	95.52	96.88	92.71
EGEUNet [54]	87.49	93.33	95.64	96.55	93.76
UL-VMUNet [26]	87.70	93.44	95.76	97.09	92.98
C2SDG [57]	88.08	93.66	95.92	97.49	92.66
HMTUNet [58]	89.19	94.28	96.60	97.39	94.74
HVMUNet [27]	89.29	94.34	96.35	97.75	93.47
VMUNet [5]	88.22	93.74	96.27	97.16	94.17
VMUNet-v2 [23]	88.87	94.11	96.15	96.92	94.54
ASP-VMUNet [4]	91.57	95.60	97.13	97.77	95.80
HC-Mamba [59]	88.73	94.03	96.45	97.32	94.37
CCViM [10]	88.86	94.10	96.47	97.06	95.06
**LTPNet** (Ours)	**92.52**	**96.12**	**97.69**	**98.22**	96.43

**Table 2:** Comparison of different models of the ISIC16 dataset. Bold values represent the best results, while underlined values represent the second-best results. ‘↑’ indicates that higher values are better.

Model	mIoU (%) ↑	DSC (%) ↑	Acc (%) ↑	Spe (%) ↑	Sen (%) ↑
UNet [12]	82.77	90.58	94.72	96.63	89.88
TransUNet [18]	86.78	92.92	95.96	96.79	93.25
MALUNet [53]	83.59	91.06	94.98	96.69	90.64
EGEUNet [54]	83.77	81.17	95.03	96.61	91.02
UL-VMUNet [26]	84.30	91.48	95.19	96.60	91.62
C2SDG [57]	84.82	91.78	95.38	96.88	91.57
HMTUNet [58]	83.13	90.79	94.84	96.69	90.14
HVMUNet [27]	85.88	92.40	95.72	97.10	92.21
VMUNet [5]	82.45	90.38	94.62	96.60	89.58
VMUNet-v2 [23]	85.91	92.42	95.70	96.72	93.10
ASP-VMUNet [4]	86.93	93.01	96.01	96.77	**94.09**
HC-Mamba [59]	85.04	91.92	95.42	96.66	92.27
CCViM [10]	85.78	92.35	95.72	**97.40**	91.45
**LTPNet** (Ours)	**87.22**	**93.18**	**96.14**	97.21	93.41

**Table 3:** Comparison of different models on the ISIC17 dataset. Bold values represent the best results, while underlined values represent the second-best results. ‘↑’ indicates that higher values are better.

Model	mIoU (%) ↑	DSC (%) ↑	Acc (%) ↑	Spe (%) ↑	Sen (%) ↑
UNet [12]	76.78	86.80	95.66	97.64	85.80
TransUNet [18]	77.17	87.11	95.83	98.17	84.17
MALUNet [53]	72.34	83.95	92.57	95.74	82.31
EGEUNet [54]	72.45	84.02	92.76	96.51	80.63
UL-VMUNet [26]	71.31	83.25	92.57	97.02	78.19
C2SDG [57]	72.79	84.25	93.00	97.22	79.35
HMTUNet [58]	70.19	82.48	92.40	97.52	75.83
HVMUNet [27]	78.36	87.87	95.96	97.67	87.45
VMUNet [5]	76.05	86.39	95.60	98.03	83.50
VMUNet-v2 [23]	73.58	84.79	93.04	96.44	82.06
ASP-VMUNet [4]	77.45	87.29	95.86	98.07	84.90
HC-Mamba [59]	77.06	87.05	95.77	97.97	84.84
CCViM [10]	78.65	88.05	96.05	97.87	86.97
**LTPNet** (Ours)	**81.04**	**89.53**	**96.55**	**98.26**	**88.04**

**Table 4:** Comparison of different models on the ISIC18 dataset. Bold values represent the best results, while underlined values represent the second-best results. ‘↑’ indicates that higher values are better.

Model	mIoU (%) ↑	DSC (%) ↑	Acc (%) ↑	Spe (%) ↑	Sen (%) ↑
UNet [12]	78.36	87.87	94.14	96.35	87.25
TransUNet [18]	77.76	87.49	93.87	95.74	88.07
MALUNet [53]	77.72	87.46	93.01	95.30	87.12
EGEUNet [54]	77.96	87.61	93.12	95.55	86.89
UL-VMUNet [26]	77.60	87.39	92.99	95.37	86.84
C2SDG [57]	77.96	87.61	93.16	95.77	86.45
HMTUNet [58]	77.25	87.16	93.09	96.69	83.82
HVMUNet [27]	79.77	88.75	94.53	96.41	88.67
VMUNet [5]	78.10	87.70	93.90	95.36	89.36
VMUNet-v2 [23]	78.19	87.76	93.04	94.53	89.20
ASP-VMUNet [4]	80.32	89.09	93.83	95.33	89.97
HC-Mamba [59]	78.62	88.03	94.24	96.56	87.03
CCViM [10]	79.87	88.81	94.55	96.40	88.82
**LTPNet** (Ours)	**82.54**	**90.43**	**95.34**	**96.91**	**90.47**

**Table 5:** Efficiency comparison on the ISIC18 dataset. ‘↑’ and ‘↓’ indicate that higher and lower values are better, respectively.

Methods	UNet	TransUNet	MALUNet	EGEUNet	UL-VMUNet	C2SDG	HMTUNet
Params (M) ↓	17.26	105.32	0.18	0.05	0.05	22.01	60.36
FLOPs (G) ↓	40.19	32.23	0.08	0.07	0.06	7.97	25.06
FPS ↑	301.75	42.11	73.55	138.44	82.24	298.53	52.24
Methods	HVMUNet	VMUNet	VMUNet-v2	ASP-VMUNet	HC-Mamba	CCViM	Ours
Params (M) ↓	8.97	35.90	22.77	0.29	13.88	32.16	26.13
FLOPs (G) ↓	0.74	5.96	4.40	0.10	2.30	4.57	4.38
FPS ↑	19.67	66.33	83.33	15.35	65.88	39.58	42.85

**Table 6:** Ablation results of key components on the PH2 dataset. Mean and standard deviation are calculated over 5 runs with different random seeds. ‘✓’ indicates the component is used. ‘↑’ indicates higher values are better. Blue indicates improvement, and red indicates performance drop.

FBA	ASM	LALGA	TPFF	mIoU ↑	DSC ↑	Acc ↑	Spe ↑	Sen ↑
				91.01 ± 0.09	95.25 ± 0.11	96.11 ± 0.12	97.73 ± 0.11	95.02 ± 0.09
✓				91.24 ± 0.15 (+0.23)	95.59 ± 0.12 (+0.34)	96.09 ± 0.08 (−0.02)	97.71 ± 0.10 (−0.02)	95.23 ± 0.14 (+0.21)
✓	✓			91.99 ± 0.13 (+0.75)	95.46 ± 0.10 (−0.13)	96.61 ± 0.12 (+0.52)	98.01 ± 0.09 (+0.30)	95.72 ± 0.11 (+0.49)
✓	✓	✓		91.95 ± 0.14 (−0.04)	95.92 ± 0.11 (+0.46)	97.37 ± 0.09 (+0.76)	97.79 ± 0.12 (−0.22)	95.69 ± 0.10 (−0.03)
✓	✓	✓	✓	92.52 ± 0.12 (+0.57)	96.12 ± 0.10 (+0.20)	97.69 ± 0.08 (+0.32)	98.22 ± 0.09 (+0.43)	96.43 ± 0.12 (+0.74)
	✓	✓	✓	92.23 ± 0.11 (−0.29)	95.98 ± 0.09 (−0.14)	97.59 ± 0.10 (−0.10)	98.17 ± 0.07 (−0.05)	96.31 ± 0.09 (−0.12)

**Table 7:** Ablation results of key components on the ISIC16 dataset. Mean and standard deviation are calculated over 5 runs with different random seeds. ‘✓’ indicates the component is used. ‘↑’ indicates higher values are better. Blue indicates improvement, and red indicates performance drop.

FBA	ASM	LALGA	TPFF	mIoU ↑	DSC ↑	Acc ↑	Spe ↑	Sen ↑
				86.72 ± 0.11	92.62 ± 0.14	95.64 ± 0.12	96.78 ± 0.09	92.89 ± 0.13
✓				86.75 ± 0.12 (+0.03)	92.48 ± 0.12 (−0.14)	95.76 ± 0.10 (+0.12)	96.89 ± 0.11 (+0.11)	92.87 ± 0.13 (−0.02)
✓	✓			86.70 ± 0.14 (−0.05)	92.39 ± 0.10 (−0.09)	95.86 ± 0.09 (+0.10)	97.14 ± 0.12 (+0.25)	92.73 ± 0.11 (−0.14)
✓	✓	✓		87.01 ± 0.11 (+0.31)	92.97 ± 0.09 (+0.58)	96.04 ± 0.10 (+0.18)	97.23 ± 0.10 (+0.09)	93.25 ± 0.12 (+0.52)
✓	✓	✓	✓	87.22 ± 0.12 (+0.21)	93.18 ± 0.10 (+0.21)	96.14 ± 0.09 (+0.10)	97.21 ± 0.11 (−0.02)	93.41 ± 0.10 (+0.16)
	✓	✓	✓	86.82 ± 0.15 (−0.40)	92.95 ± 0.12 (−0.23)	96.01 ± 0.08 (−0.13)	97.14 ± 0.07 (−0.07)	93.15 ± 0.12 (−0.26)

**Table 8:** Ablation results of key components on the ISIC17 dataset. Mean and standard deviation are calculated over 5 runs with different random seeds. ‘✓’ indicates the component is used. ‘↑’ indicates higher values are better. Blue indicates improvement, and red indicates performance drop.

FBA	ASM	LALGA	TPFF	mIoU ↑	DSC ↑	Acc ↑	Spe ↑	Sen ↑
				78.57 ± 0.07	88.00 ± 0.06	96.10 ± 0.09	98.27 ± 0.08	86.93 ± 0.07
✓				78.72 ± 0.16 (+0.15)	88.09 ± 0.15 (+0.09)	96.08 ± 0.12 (−0.02)	98.21 ± 0.10 (−0.06)	87.23 ± 0.14 (+0.30)
✓	✓			79.57 ± 0.15 (+0.85)	88.12 ± 0.13 (+0.03)	96.32 ± 0.10 (+0.24)	97.98 ± 0.11 (−0.23)	87.68 ± 0.11 (+0.45)
✓	✓	✓		80.59 ± 0.14 (+1.02)	88.99 ± 0.12 (+0.87)	96.10 ± 0.11 (−0.22)	98.27 ± 0.09 (+0.29)	87.95 ± 0.10 (+0.27)
✓	✓	✓	✓	81.04 ± 0.12 (+0.45)	89.53 ± 0.10 (+0.54)	96.55 ± 0.09 (+0.45)	98.26 ± 0.11 (−0.01)	88.04 ± 0.12 (+0.09)
	✓	✓	✓	79.98 ± 0.08 (−1.06)	88.76 ± 0.07 (−0.77)	96.41 ± 0.10 (−0.14)	98.05 ± 0.09 (−0.21)	87.86 ± 0.07 (−0.18)

**Table 9:** Ablation results of key components on the ISIC18 dataset. Mean and standard deviation are calculated over 5 runs with different random seeds. ‘✓’ indicates the component is used. ‘↑’ indicates higher values are better. Blue indicates improvement, and red indicates performance drop.

FBA	ASM	LALGA	TPFF	mIoU ↑	DSC ↑	Acc ↑	Spe ↑	Sen ↑
				80.21 ± 0.15	89.12 ± 0.11	93.88 ± 0.13	96.02 ± 0.10	89.24 ± 0.14
✓				80.20 ± 0.13 (−0.01)	89.15 ± 0.12 (+0.03)	93.75 ± 0.14 (−0.13)	96.19 ± 0.11 (+0.17)	89.20 ± 0.13 (−0.04)
✓	✓			80.85 ± 0.14 (+0.65)	90.12 ± 0.13 (+0.97)	94.53 ± 0.12 (+0.78)	96.13 ± 0.10 (−0.06)	89.89 ± 0.12 (+0.69)
✓	✓	✓		81.69 ± 0.15 (+0.84)	90.09 ± 0.12 (−0.03)	94.89 ± 0.11 (+0.36)	96.37 ± 0.13 (+0.24)	90.01 ± 0.10 (+0.12)
✓	✓	✓	✓	82.50 ± 0.08 (+0.81)	90.42 ± 0.10 (+0.33)	95.34 ± 0.09 (+0.45)	96.92 ± 0.11 (+0.55)	90.46 ± 0.08 (+0.45)
	✓	✓	✓	81.76 ± 0.18 (−0.74)	90.21 ± 0.11 (−0.21)	95.13 ± 0.17 (−0.21)	96.55 ± 0.13 (−0.37)	90.35 ± 0.14 (−0.11)

**Table 10:** Ablation results of TPFF path on the ISIC18 dataset. ‘✓’ indicates the component is used. ‘↑’ indicates higher values are better. Blue indicates improvement, and red indicates performance drop.

CP	SP	DP	mIoU (%) ↑	DSC (%) ↑	Acc (%) ↑	Spe (%) ↑	Sen (%) ↑
✓			82.12	90.13	95.09	96.79	90.01
✓	✓		82.32 (+0.20)	90.50 (+0.37)	95.19 (+0.10)	96.47 (−0.32)	90.23 (+0.22)
✓	✓	✓	82.54 (+0.22)	90.43 (−0.07)	95.34 (+0.15)	96.91 (+0.44)	90.47 (+0.24)

**Table 11:** Comparison of Baseline, SOTA, and LTPNet performance with MobileMamba backbone on the PH2 dataset. ‘↑’ indicates higher values are better.

Method	Backbone	mIoU ↑	DSC ↑	Acc ↑	Spe ↑	Sen ↑
Baseline	MobileMamba	91.01	95.25	96.11	97.73	95.02
VMUNet [5]	MobileMamba	91.77	95.71	97.45	97.97	96.20
VMUNet-v2 [23]	MobileMamba	92.20	95.94	97.59	98.21	96.14
HMTUNet [58]	MobileMamba	90.24	94.87	96.96	97.86	94.82
LTPNet(Ours)	MobileMamba	92.52	96.12	97.69	98.22	96.43

**Table 12:** Comparison of different models on the CVC-ClinicDB dataset. Bold values represent the best results, while underlined values represent the second-best results. ‘↑’ indicates that higher values are better.

Model	mIoU (%) ↑	DSC (%) ↑	Acc (%) ↑	Spe (%) ↑	Sen (%) ↑
UNet [12]	81.73	89.95	98.29	98.98	90.83
TransUNet [18]	85.69	92.29	98.71	99.36	91.62
MALUNet [53]	39.33	81.88	96.92	98.20	82.95
EGEUNet [54]	65.13	78.88	96.45	98.06	78.93
UL-VMUNet [26]	72.52	84.07	97.35	98.64	83.28
C2SDG [57]	86.64	92.84	98.78	99.22	93.99
HMTUNet [58]	82.14	90.19	98.36	99.13	89.97
HVMUNet [27]	84.61	91.66	98.61	99.30	91.07
VMUNet [5]	81.95	90.08	98.42	99.18	89.73
VMUNet-v2 [23]	89.31	94.35	**99.08**	99.28	**95.64**
ASP-VMUNet [4]	71.40	83.31	97.17	98.39	83.92
HC-Mamba [59]	83.56	91.05	98.47	98.99	92.74
CCViM [10]	84.77	91.76	98.62	99.25	91.72
**LTPNet** (Ours)	**89.58**	**94.51**	**99.08**	**99.55**	94.00

## Data Availability

The data presented in this study are openly available in https://github.com/hpguo1982/LTPNet (accessed on 18 December 2025).
